# Discerning the impact of ctDNA detection on patient decision-making in early-stage breast cancer

**DOI:** 10.1038/s41523-024-00701-y

**Published:** 2024-10-08

**Authors:** Tarah J. Ballinger, Mary Lou Smith, Elda Railey, Greg Zimet, Bryan P. Schneider

**Affiliations:** 1 Indiana University School of Medicine, Indianapolis, IN USA; 2 Research Advocacy Network, Plano, USA

**Keywords:** Breast cancer, Prognostic markers

## Abstract

The impact of knowledge of circulating tumor DNA (ctDNA) status on patient decisions in high-risk triple-negative breast cancer (TNBC) weighing benefit and toxicity is unknown. Here, 286 women with a history of non-metastatic breast cancer who had received chemotherapy completed a survey mimicking scenarios of residual TNBC after chemotherapy and unknown, negative, or positive ctDNA status to determine the shift in the decision to receive adjuvant therapy. Participants were then presented scenarios mimicking possible post-neoadjuvant therapies and rated acceptability. A general linear model with repeated measures determined contributions of risk reduction and toxicity. When the hypothetical risk of recurrence mimicked ctDNA negativity, significantly less participants were accepting of adjuvant capecitabine versus no therapy. When presented with ctDNA positivity and increased recurrence risk, the degree of benefit impacted acceptability more than the toxicity profile. As genomic technology advances and ctDNA assays become commercially available, it is imperative to understand the impact on patient decision-making.

## Introduction

Breast cancer patients face complicated therapeutic decisions in the curative setting, and these decisions impact both disease and quality of life (QoL) outcomes. Prior data have suggested that patients in the curative setting are willing to accept significant toxicity for a small gain in survival benefit^1,2^. Subsequent data from our group found that patients would be willing to accept a trade-off in efficacy for the minimization of key toxicities seen with more contemporary therapies^3^. Recent advances in genomic technologies, such as minimal residual disease (MRD) detection, are becoming more accessible and may make it possible to further refine prognosis or personalize treatment recommendations. This type of information can alter patient perception of risk, and thus how risk versus benefit is weighed in making decisions that ultimately influence what therapy is received. It is imperative to understand how MRD information might impact patient decision-making.

Patients with triple-negative breast cancer (TNBC) and residual disease after neoadjuvant chemotherapy have a high risk of recurrence, and reducing recurrence risk in this space is an unmet need. Currently, a standard approach to post-neoadjuvant therapy for residual TNBC includes the continuation of adjuvant pembrolizumab per the KEYNOTE-522 trial^4^, as well as the addition of capecitabine based on a survival benefit seen in the CREATE-X trial^5^. In addition, olaparib is also FDA approved as adjuvant therapy for patients with high-risk disease and germline *BRCA1/2* mutations based on improved DFS in the OlympiA trial^6^. While the implementation of these agents improves outcome, the risk of relapse remains high and further work to improve outcomes and to better risk stratify remain critical. One strategy under investigation is to apply additional targeted therapy to standard therapy. This strategy has been evaluated in the preoperative setting with the I-SPY2 trials^7,8^. Similarly, BRE12-158 tested a personalized approach in this very high-risk population of TNBC with residual disease after standard chemotherapy^9^. In BRE12-158, patients with ctDNA positivity after neoadjuvant therapy for TNBC (MRD+) experienced a significant increase in risk of disease recurrence, while negative ctDNA was associated with a significant decrease in risk (distant disease-free survival at 2 years 56% in ctDNA positive versus 81% in ctDNA negative, HR 2.99, *p* = 0.006)^10^. Similar data has been shown in other trials, with MRD positivity being associated with markedly higher risk of recurrence^11,12^. Several recently completed or ongoing trials prospectively stratify therapy based on ctDNA and evaluate the impact of additional therapy in patients with TNBC. The c-TRAK TN trial prospectively assessed ctDNA by digital pCR in patients with residual TNBC, with a plan for staging imaging and pembrolizumab initiation for those with positivity. Implementation was limited by the significant amount of metastatic disease detected at imaging and small patient numbers^13^. The ongoing PERSEVERE trial (NCT04849364) stratifies therapy on ctDNA status, while also testing the impact of adding targeted therapy to standard therapy in the ctDNA positive arm, including immunotherapy, PARP inhibition and PIK3/AKT pathway inhibition.

Although advancements in detection of MRD and next- generation sequencing make it possible to refine prognosis and may lead to therapeutic advancements, it is not clear if, and to what degree, individual shift in prognosis and treatment options are relevant to decisions made by patients. Here, we report a ratings- based analysis to determine whether additional prognostic knowledge gained by ctDNA detection influences patient choice of therapy in the post-neoadjuvant setting using conventional regimens. Additionally, we model potential molecularly- targeted adjuvant therapies to quantify the relative influence of changing degrees of absolute benefit versus toxicity profiles on treatment choice in patients with high-risk TNBC.

## Methods

**Fig. 1:**
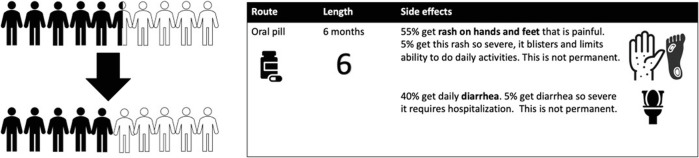
Example of graphics accompanying descriptions of clinical scenarios. Example displays a 5% absolute reduction in risk of recurrence and the toxicity profile of adjuvant capecitabine.

Patients who self-reported a history of early-stage breast cancer treated with chemotherapy at least 6 months ago but not more than 10 years ago were recruited for study participation. This population with experience with prior chemotherapy was chosen to mimic those who have experienced prior toxicity of chemotherapy, which may influence the decision of whether to accept further therapy in the post-neoadjuvant setting. Fig. 1 includes an example of how data was presented graphically to patients. Participants were recruited via email from US-based advocacy groups Living Beyond Breast Cancer, Young Survivor’s Coalition, and Pink-4-Ever Ending Disparities with the goal of ensuring sampling of young patients and Black patients, who are at disproportionate risk of TNBC^14^. The study was approved by the Indiana University Institutional Review Board. The need for informed consent was waived as no personal health information was collected.

### Survey

Following confirmation of eligibility, participants were given a one-time cross-sectional survey delivered via Qualtrics. Throughout the survey, no drug names or classes were used. Benefit was modeled in terms of absolute recurrence risk reduction and shown both numerically and visually using pictographs. Toxicity was presented with acute and potential long-term side effects, with both written description and visual icons. All scenarios were presented in random order to minimize confounders due to patient fatigue later in the survey. The survey was developed in collaboration with patient advocates and tested to ensure comfortability and understanding. Participants were provided a $25 Amazon gift card at completion.

The survey consisted of three parts. The first section presented participants with a hypothetical scenario in which they had received chemotherapy and had residual disease at the time of surgery, indicating a baseline risk of recurrence of 40%^5^. Participants then chose between scenarios mimicking toxicity and benefit of standard of care adjuvant capecitabine (based on data from ECOG-ACRIN EA1131^15^) versus no further therapy (based on CREATE-X no therapy arm^5^). The situation was then altered to mimic the same scenario but knowledge of ctDNA negative status and thus a risk of recurrence decreased to 20% based on data from BRE12-158^10^. The risk profile estimates for ctDNA positivity or negativity used in this hypothetical survey study were based on tissue-agnostic platforms and thus do not reflect the sensitivity and specificity of some of the tissue-informed platforms used at the time of this publication.

The second section was designed to mimic a scenario in which patients knew they were ctDNA positive, and thus had a risk of recurrence of 55% based on data from BRE12158^10^. Participants were then presented with scenarios varying among two dimensions: 1) toxicity with four attribute levels mimicking standard of care and potential genomically-guided therapies (capecitabine, PARP inhibition, immunotherapy, and PI3K/AKT inhibition), and 2) benefit with three attribute levels for absolute risk reduction of therapy (5%, 15%, or 35%). Participants were asked to provide a likelihood that they would accept a combination of toxicity and benefit on a scale from 0 (definitely not acceptable) to 100 (definitely acceptable).

Patients lastly provided basic demographics, socioeconomic status, past breast cancer history, their perceived actual risk of recurrence, and experience with prior toxicities. This section was delivered last so that the participants are least fatigued when answering the actual study questions.

### Statistical analysis

We aimed to recruit at least 200 participants. A sample size of 200 is sufficient for ratings-based analysis, which is a within-subjects design that maximizes statistical power and is comparable to sample sizes used in other ratings-based analyses. Descriptive statistics summarized demographic data continuously or categorically. In section 1 of the survey, the number of participants agreeing to further chemotherapy was compared in each scenario using McNamar’s Chi-square test to account for repeated measures. In section 2 of the survey, participants were given a total of 12 scenarios (representing four toxicity attributes and three benefit attributes), representing a full factorial design (i.e. all possible combinations, allowing for evaluation of the main effects of the attributes). A general linear regression analysis with repeated measures was performed using SPSS v.28. The regression separates the overall likelihood rating (i.e. utility) into parts depending on the relative importance of each attribute’s level. The beta weights from the regression model represent part-worth utilities of each attribute and measure how patients value toxicity versus benefit when making decisions about further therapy in this setting. A negative part-worth utility represents relative dislike for an attribute, while a positive value represents preference of an attribute. Attributes with the largest part-worth utility are the most important contributors to preference. A priori subgroups of interest, including age (<40, ≥40), stage of cancer (I, II/III), prior experience with chemotherapy toxicity (Yes, No), and current chemotherapy toxicity (Yes, No) were evaluated to examine whether preferences differed using McNamar’s chi-square test. Prior toxicity was measured based on self-report of significant side effects that led to dose reductions or dose discontinuation; current toxicity was indicated by residual effects the participant attributes to chemotherapy (i.e. fatigue, cognitive dysfunction, peripheral neuropathy, cardiac issues, etc.).

## Results

### Respondent demographics

286 respondents were eligible and completed the survey with evaluable responses in April and May of 2023. The survey took an average of 15.4 min to complete. Average age was 41.2 (range 2775) and patients were an average of 4.5 years out from their breast cancer diagnosis. Almost all respondents were from Young Survivors’ Coalition (Table 1).

**Table 1:** Participant characteristics

Variable	% (N)
Age (median, range)	41.2 (27–75)
Race	
White	82.5 (236)
Black	8.7 (25)
Asian	10 (3.5)
Other	15 (5.2)
Organization	
Young Survivor’s Coalition	93.4 (267)
Living Beyond Breast Cancer	5.6 (13)
Pink-4-Ever Ending Disparities	2.1 (6)
Education	
Less than high school	0.1 (2)
High school	10.8 (31)
College degree	40.2 (115)
Graduate degree	47.9 (137)
Unknown	1
Health insurance status	
Insured	97.2 (278)
Uninsured	1.4 (4)
Unknown	1.4 (4)
Household Income	
Less than 50k	9.0 (26)
50-90k	18.9 (54)
90-120k	18.2 (52)
Over 120k	45.5 (130)
Unknown	8.4 (24)
Disease stage at diagnosis	
I	27.6 (79)
II	49.3 (141)
III	23.1 (66)
Prior toxicity to therapy	
Yes	41.6 (119)
No	58.3 (167)
Current toxicity from prior therapy	
Yes	82.9 (237)
No	17.1 (49)

### Impact of ctDNA status on post-neoadjuvant therapy decision

**Fig. 2:**
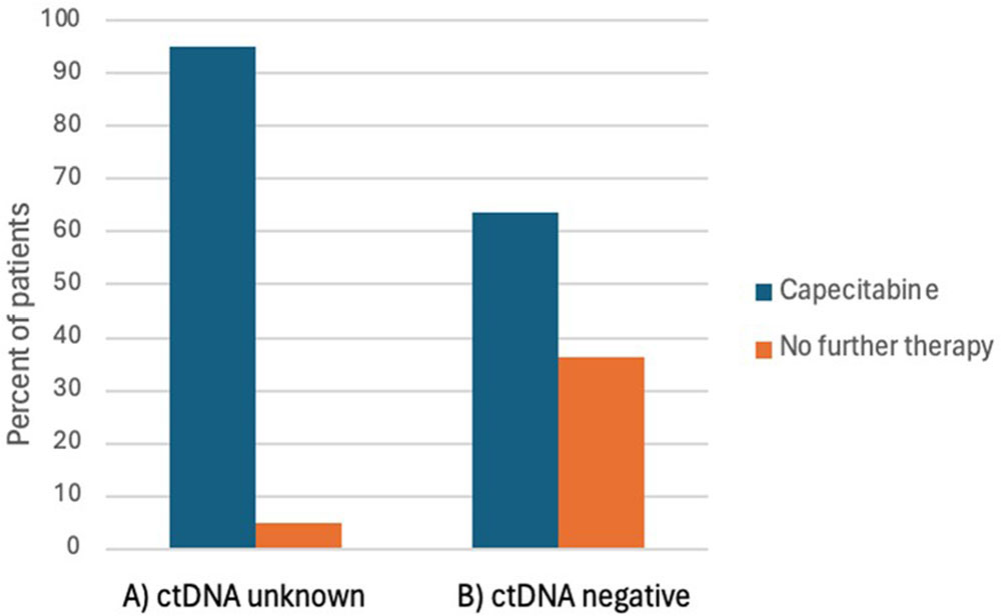
Proportion of patients accepting a scenario mimicking adjuvant capecitabine in the setting of residual disease after neoadjuvant chemotherapy, compared to no further therapy. (**A**) is preference when ctDNA status is unknown, and (**B**) is shift in preference with knowledge of negative ctDNA status (shift significant at *p* < 0.001).

When risk of recurrence was decreased from 40% to 20%, mimicking negative ctDNA status, significantly less participants indicated preference for a medication similar to adjuvant capecitabine over no further therapy (95.1% versus 63.6%, *p* < 0.001). Approximately one-third (*n* = 91, 31.8%) shifted preference away from further therapy with the knowledge of ctDNA negativity (Fig. 2). 181 respondents (63.3%) chose capecitabine over no further therapy in both scenarios, regardless of risk of recurrence. 13 participants (4.5%) chose not to receive capecitabine in either scenario; only one chose capecitabine when risk was 20%, but not at 40%, potentially reflecting a lack of understanding or a mistake. This pattern of responses was similar regardless of age, cancer stage, and chemotherapy toxicity experience.

### Post-neoadjuvant therapy acceptability

When presented with a risk of recurrence of 55%, mimicking positive ctDNA status, both benefit and toxicity profile significantly influenced acceptability with preference for higher risk reduction and lower toxicity (benefit: *F* = 448.5, *p* < 0.001; toxicity: *F* = 33.64, *p* < 0.001). Benefit had a larger effect size on acceptability, compared to toxicity (η^2^ = 0.76 and 0.26, respectively). There were no statistically significant interactions between age, stage of cancer, or prior or present chemotherapy toxicity with the impact of toxicity profile or with benefit on treatment acceptability ratings (all *p*-values > .30). The most preferred regimen overall was one that mimicked one year of PARP inhibitor therapy with a 35% absolute risk reduction (mean acceptability rating 87.8/100). However, if the PARP inhibitor therapy only had a 15% risk reduction, then preference shifted to any of the other regimens with a higher benefit. Overall, respondents favored maximum risk reduction regardless of the toxicity profile of the regimen. Even when presented with only a 5% absolute reduction in risk, treatment acceptability rating was still 31.5 across all toxicity profiles, indicating many patients in this setting would have accepted further therapy for even a small benefit (Table 2).

**Table 2:** Acceptability rating for toxicity profiles of possible genomically- directed postneoadjuvant treatment regimens with varying degrees of benefit

Toxicity profile	Absolute risk reduction	Mean score	Std Dev
A (capecitabine)	5	38.84	32.21
A (capecitabine)	15	57.40	31.10
A (capecitabine)	35	86.28	20.43
B (immunotherapy)	5	32.89	30.00
B (immunotherapy)	15	53.31	29.47
B (immunotherapy)	35	82.0	22.54
C (parp inhibitor)	5	43.22	34.45
C (parp inhibitor)	15	62.12	30.53
C (parp inhibitor)	35	87.71	21.56
D (PI3K inhibitor)	5	30.80	29.46
D (PI3K inhibitor)	15	51.28	31.09
D (P inhibitor)	35	78.20	25.28

## Discussion

Patient decision-making for therapy selection is based on innumerable factors, heavily considering perceived baseline risk of recurrence, and the potential benefit and toxicity conferred by a given therapy. Patients with TNBC who have residual disease after standard pre-operative therapy have a high risk for recurrence and cancer-specific death. Continuation of pembrolizumab, capecitabine, and/or olaparib (for germline *BRCA1/2* mutation carriers) have all been shown to improve outcomes in this setting^4–6^; each has limitations and unique toxicity risk, and the impact of overlapping or sequencing these therapies has not yet been realized. More recent data has shown that MRD positivity further refines risk in this setting, with patients who are ctDNA positive having an exceptionally high risk of recurrence^10–12^. Based on these data gaps and the high risk for recurrence, multiple trials are planned and/or ongoing to improve outcomes in this space. These patients have already completed a significant amount of therapy in the pre-operative setting; the complexity of navigating a high risk of recurrence with the toxicity of additional therapy can be a substantial burden. Understanding the patient perspective and what results would be clinically impactful are critical to properly planning and interpreting future advances in this setting.

Here, we demonstrate that the knowledge of an additional prognostic indicator, such as the information provided by ctDNA status, significantly altered the proportion of patients who felt additional therapy was acceptable. We modeled capecitabine as standard of care therapy to reduce risk of recurrence in patients with residual TNBC, based on the CREATE-X trial^5^. ‘Real-world’ data on the uptake of standard of care capecitabine therapy in the setting of residual disease is lacking. However, our analysis demonstrated that one third of patients would shift away from accepting capecitabine if their risk of recurrence was lower based on ctDNA negativity. This suggests that patients benefit from individualized prognosis and toxicity information to optimize decision making. Interestingly, patient acceptability of post-neoadjuvant therapy was not modulated by the age of the patient, stage of their disease, or whether they had experienced toxicity with prior chemotherapy. We were not able to discern from the data available any characteristics that were associated with being more or less accepting of post-neoadjuvant therapy; reinforcing how multivariate and individualized these decisions are.

In this study, we modeled potential standard of care or genomically- directed therapies that may be delivered in the post-neoadjuvant setting based on ongoing clinical trials. Our survey study supports a high acceptability of these therapies, including PARP inhibition, immunotherapy, and PIK3/AKT pathway inhibitors, even if small benefit, in patients who have a higher risk of recurrence based on positive ctDNA status. Similar to prior work in the curative setting around standard chemotherapy treatment options, when faced with tradeoffs between toxicity and benefit, patients weighed the change in benefit more substantially than toxicity profiles when determining acceptability^1–3^. The ratings-based analysis carried out here indicates that patients will accept any of the potential regimens outlined for a substantial absolute reduction in recurrence risk. The highest acceptability level was for an oral medication with a side effect profile most similar to a PARP inhibitor. Even with only a 5% absolute benefit, the PARP inhibitor model still received an acceptability score of 43.2/100.

In current practice, while not guideline-based, patients can receive ctDNA results with some commercially available assays. Here, our analysis indicates that patients would shift decision on receiving potentially curative therapy based on these results. Importantly, however, the desire to accept additional therapy is under the assumption that there is an increased absolute benefit in cure from these therapies for those who are ctDNA positive due to a higher risk of recurrence. Unfortunately, while the goal of existing therapies and ongoing trials is to further reduce risk in this space, there is no current evidence that therapeutic interventions in the ctDNA positive population improve outcomes. Thus far, no study has shown that intervening on MRD positivity improves outcomes, or that de-escalating or stopping therapy for MRD negativity is safe. In addition, the utility of these assays is impacted by their sensitivity, which is variable and becomes more accurate with repeated testing using ultra-sensitive, tumor-informed assays^12,16^. Thus, results of these tests must be interpreted with caution until trials have demonstrated clinical utility. While it is appropriate to assume risk reduction is most important to patients when risk of recurrence is high, we must have effective tools to complete the equation.

Beyond potential influence on therapy decisions without predictive utility, ctDNA status may have impact on patient QoL. Prior work has found unrealistic patient expectations for participation in genomic sequencing studies in patients with metastatic disease^17^. However, high levels of satisfaction and low levels of decisional regret in this setting suggest this may not be overly harmful. In contrast, moving these misunderstandings to the curative setting may have more significant and longer- term implications. Not captured in our analysis is how knowledge of ctDNA status might impact not only patient decision-making, but other patient-reported outcome measures as well, including QoL, anxiety, and fear of cancer recurrence. Fear of cancer recurrence is a prevalent, long-term experience among breast cancer survivors, associated with higher anxiety, lower QoL, and higher health-care costs^18^. While prior work has suggested that actual risk is not associated with fear of recurrence^19,20^, it is unknown how the tangible results of MRD testing will impact these symptoms in women living the experience. Ongoing and developing trials should include patient-reported outcome measures to assess the impact of receiving ctDNA results, such as preference for receiving results, fear of cancer recurrence index (FCRI), assessment of survivors’ concerns (ACS), impact of events scale, PROMIS-29 health-related QoL, PROMIS anxiety short form, and the breast cancer self-efficacy scale^21–23^.

There are an innumerable number of factors that contribute to patient decision making that cannot be captured and analyzed in a survey analysis. However, aggregating responses from a large group of patients with prior chemotherapy experience allows us to summarize how the altered prognostic information provided by ctDNA status shifts choice. The population of patients enrolled in this study had received prior chemotherapy and had not yet reoccurred; therefore, it is possible that they are biased toward the benefit of therapy versus toxicity. Our survey does not capture the participants’ actual nor perceived risk of recurrence. While we provided risk estimates in the survey and asked patients to think about those numbers, it is possible patients’ own perception of their risk influenced choice. A further inherent limitation in this type of hypothetical analysis is that we do not know how the discussion with a physician would influence a patient’s choice. As novel assays become more accessible, it will be increasingly important that patients understand what test results mean and what they do not mean. Just as many variables enter a decision from the patient perspective, physicians weigh numerous factors when ordering and interpreting a test^24^, and efforts to ensure physicians accurately understand the utilities of MRD assays are paramount. Patients are also exposed frequently to information regarding novel technologies in the media or social platforms that is not always accurate, and may influence decisions. In addition, selection bias is a relevant weakness of this study. Despite an attempt to over-sample Black patients due to disproportionate prevalence of TNBC, they represented only 8% of the population sampled. The survey population was also highly education and nearly all had health insurance. This not only limits generalizability of responses, but also does not allow us to determine the degree to which race or socioeconomic factors influenced choice in this hypothetical setting. It is well established that social determinants and social risks influence decision making, and may lead to favoring an economic or social benefit over a disease-related risk from forgoing expensive therapies and additional appointments^25^.

Importantly, this study highlights that patient decision-making is altered based on additional prognostic information in the high risk setting of residual TNBC. This is likely true not just of this model of MRD, but of other prognostic markers beyond ctDNA as well. However, there is a striking lack of information on the impact of MRD detection and genomic information on patient-reported outcomes in the curative setting for breast cancer. Patient advocates and patients’ voices should be included in the design of trials that incorporate detection of MRD. When considering the clinical utility of a prognostic or predictive assay, impact on the patient and on treatment decision-making should be part of that equation. Future work should determine short and long-term associations of ctDNA detection with QoL, anxiety, fear of cancer recurrence, and unnecessary testing. Further work is also needed to determine the optimal ways to return this type of information to the patient in way that is both sensitive and informative.

## Data Availability

A minimal dataset of survey responses is not publicly available but is available upon approved request from the corresponding author.
